# Asymptomatic Intracardiac Cement Embolism Following Kyphoplasty

**DOI:** 10.7759/cureus.38735

**Published:** 2023-05-08

**Authors:** Aakash A Setty, David C Gimarc, Barbara Abrahams, Corey K Ho

**Affiliations:** 1 Radiology, Warren Alpert Medical School of Brown University, Providence, USA; 2 Radiology, University of Colorado Anschutz Medical Campus, Aurora, USA; 3 Cardiology, University of Colorado Anschutz Medical Campus, Aurora, USA

**Keywords:** intra-cardiac cement, anticoagulation, cardiac cement, cement embolization, proximal junctional kyphosis, vertebral augmentation, percutaneous vertebroplasty, kyphoplasty, polymethyl methacrylate (pmma), cement leakage

## Abstract

Cement extravasation can occur during vertebral body augmentation such as kyphoplasty and vertebroplasty with diverse presentation and resultant treatment. The cement can embolize through venous vasculature to the thorax where it poses a potential threat to the cardiovascular and pulmonary systems. A thorough risk-benefit analysis should be conducted to select the appropriate treatment course. We present an asymptomatic case of cement extravasation to the heart and lungs during kyphoplasty.

## Introduction

Vertebral augmentation is a common percutaneous procedure in the treatment of vertebral compression fractures, often due to primary and secondary osteoporosis, neoplasm, and trauma. This includes conventional vertebroplasty and kyphoplasty, which involves cavity creation before filling it with cement. Newer techniques also utilize device implantation in combination with cement fixation. The cement polymer is composed of polymethylmethacrylate, which enters as a viscous paste and hardens over time as it cures. These procedures can reduce back pain and restore stability to the spinal column [1], thereby often reducing the usage of opioids and analgesics and improving functional outcomes.

There are risks involved with vertebral augmentation. Cement may leak out of the vertebral body during the procedure into the venous outflow, epidural space, disk space, or neural foramen, of which a small fraction may develop symptoms or necessitate further intervention. The reported rates of cement leakage vary widely in literature: A systematic review reported an estimated rate of cement leakage of 18.1% for kyphoplasty and 41.1% for vertebroplasty [2]. This case presents venous cement extravasation leading to asymptomatic intracardiac embolization. This adds to the existing literature on decision-making for treating cement leakage during vertebral augmentation and also demonstrates a possible pre-procedural indicator for the proclivity of cement extravasation.

## Case presentation

**Figure 1:**
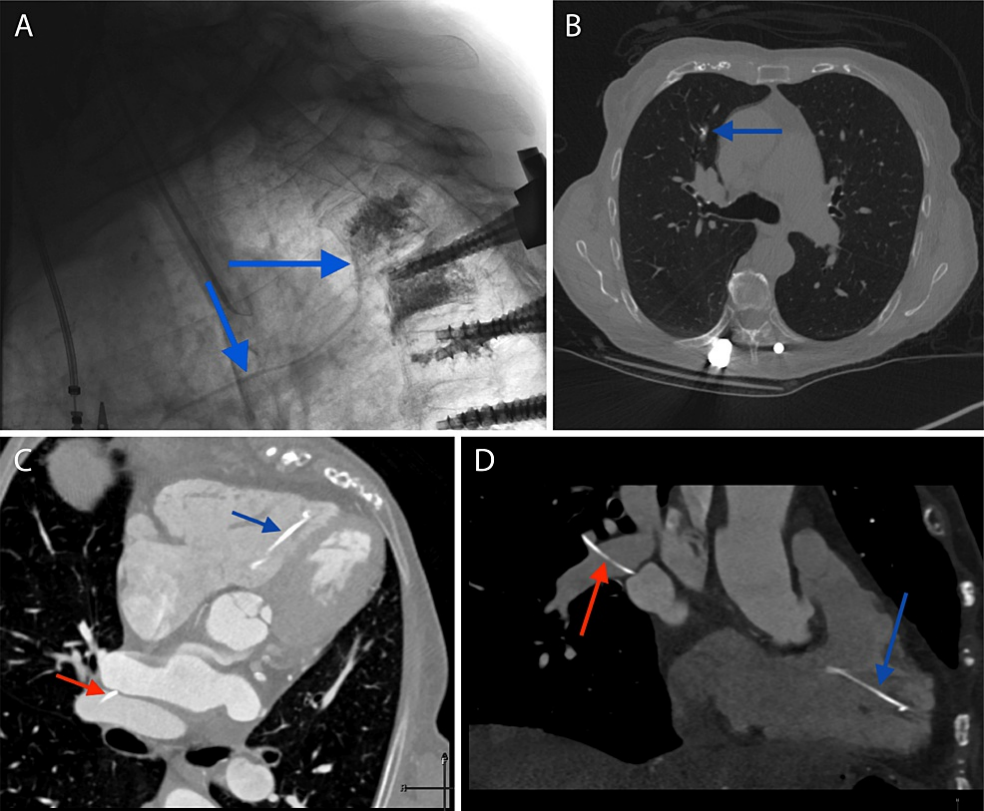
Fluoroscopy and CT imaging depicting cement extravasation and emboli locations (A) The sagittal intra-procedural fluoroscopic image shows radiodense cement emanating from the T3 vertebral body toward the thorax (blue arrows). An endotracheal tube and posterior spinal fusion hardware are partially in the field of view. (B) An axial CT of the chest shows a small focus of cement within a segmental arterial branch of the middle lobe of the right lung (blue arrow). (C) An axial oblique CT image of the heart demonstrates a linear focus of cement within the right ventricle (blue arrow). Some linear cement is seen along the right pulmonary artery as well (red arrow). (D) A coronal oblique CT image of the heart demonstrates a linear focus of cement within the right ventricle (blue arrow). Some linear cement is seen along the right pulmonary artery as well (red arrow).

A 78-year-old woman with existing spinal fusion extending from T4 through the pelvis presents with proximal junctional level breakdown/adjacent segment disease including endplate fracturing of her T3 and T4 vertebral bodies. Kyphoplasty was performed at T3 and T4 through a unipedicular and trans-discal approach, with cement extravasation visualized along anterior paravertebral veins extending to the mediastinum during the procedure (Figure 1, Panel A). A standard cement mix was used following the manufacturer's mixing instructions. Although the patient was asymptomatic, a chest CT was performed due to the cement pattern seen during the procedure. There was cement extravasation extending from the paravertebral veins with foci nearly to the azygos vein, within the right ventricle, and within the right pulmonary artery with more distal pulmonary cement emboli (Figure 1, Panels B-D).

**Figure 2:**
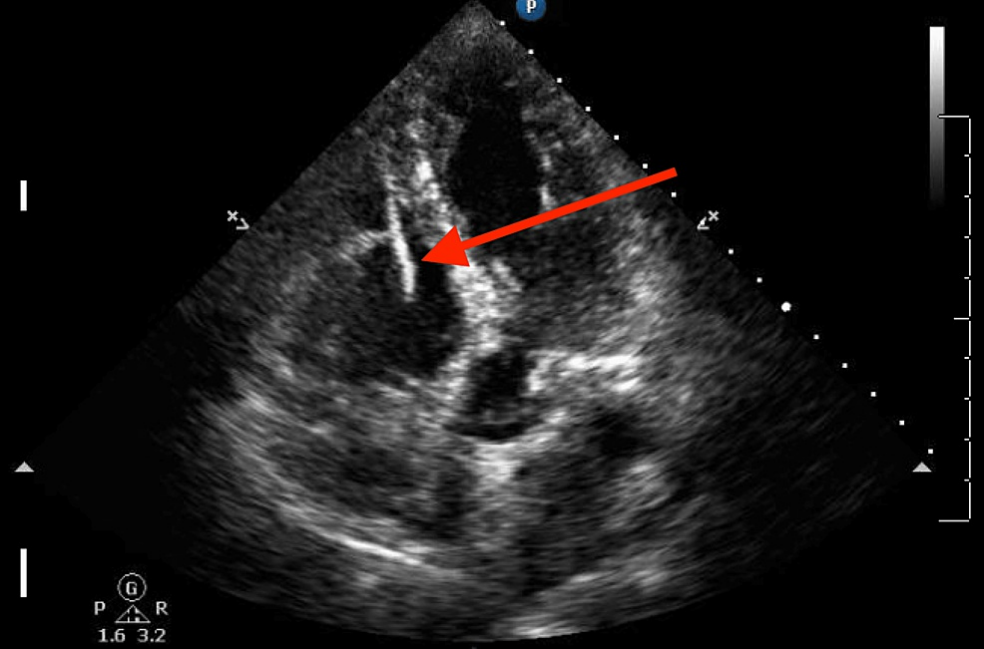
Transthoracic echocardiogram of cement embolus Transthoracic echocardiogram shows a linear, non-flexing, and immobile structure extending from the tricuspid annulus to nearly the right ventricular apex (red arrow). No adherent mass is seen.

The intracardiac cement focus was confirmed by transthoracic echocardiography (Figure 2).

**Figure 3:**
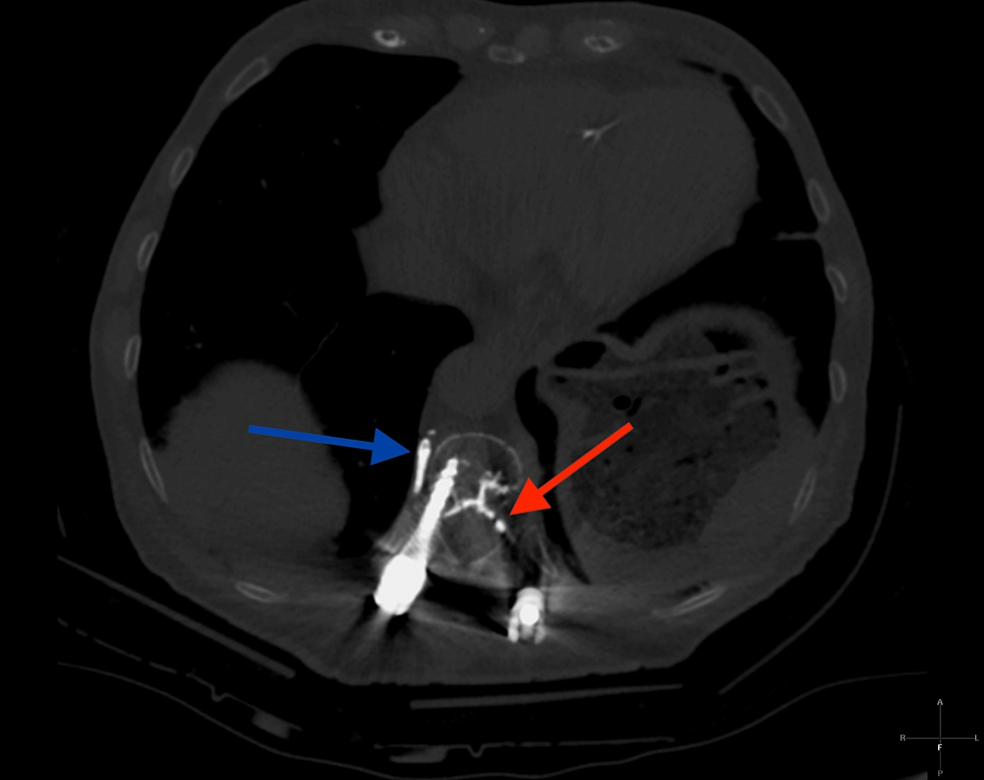
CT imaging of previous cement extravasation An axial CT image of the chest demonstrates extravasated cement from the prior spinal fusion surgery. This is seen in a paravertebral vein (blue arrow) and along Batson’s plexus extending to the epidural space (red arrow).

Cement extravasation from prior spinal fusion was also seen scattered along the epidural space, extending from lower levels of the existing orthopedic fusion hardware (Figure 3).

Prior to the kyphoplasty procedure, the patient had a 60-year history of recurrent vasovagal syncope, occasional to frequent premature ventricular contractions (PVC), and premature atrial contractions (PAC) as well as short runs of asymptomatic ventricular and supraventricular tachycardia, frailty, and Parkinson's disease. She had no pacemaker or implantable cardioverter-defibrillator. Despite the imaging findings, a combination of consultation with cardiology, a lack of literature that suggested obligatory cement removal, and the preference of the patient to avoid prophylactic cement removal resulted in the decision for clinical follow-up rather than surgical intervention. The patient was not initiated on anticoagulant therapy.

Due to the patient's wishes and lack of symptoms, a follow-up was performed at eight months. Transthoracic echocardiography redemonstrated the cement embolism without change or new thrombus. At extended follow-up 24 months following the procedure, the patient remains asymptomatic without the need for cardiothoracic intervention.

## Discussion

Although the cause of cement leakage is multifactorial in cement augmentation procedures, it has been determined to correlate most strongly with fracture anatomy, cement viscosity, and cement volume in a prior meta-analysis [3]. Common locations of cement leakage include intervertebral disc space, paraspinal venous circulation, and epidural space. In our patient, the unhardened cement traveled via a paravertebral vein to the azygos vein. The additional cement found in the right ventricle may have hardened in the venous circulation and embolized to the heart via the azygous and superior vena cava. Additional foci were seen in the pulmonary circulation.

Previous cement leakage has been presented in the literature featuring both asymptomatic and symptomatic presentation with medical management or cement retrieval surgery (embolectomy). Cardiac symptoms when present can include sudden or progressive onset of angina and/or dyspnea, elevated troponin, and electrocardiography findings including tachycardia and ST-segment elevations [4,5]. The literature presents numerous cement leakage cases requiring embolectomy including myocardial perforation with pericardial tamponade treated with open-heart embolectomy, nerve root impingement treated with spinal decompression and open-spine embolectomy, and pleural perforation treated with open-thoracic embolectomy [6-8].

The right ventricular embolus shape in this patient is similar to other embolized cement “spears” found in both asymptomatic cases and symptomatic cases requiring embolectomy [4,5,8,9]. In previous cases featuring a cement spear in the right ventricle and no subsequent embolectomy, the patients remained asymptomatic over several years after multidisciplinary consultation and a risk-benefit analysis [10,11]. Cases have also been presented with medically managed treatment of symptoms discovered days to weeks after surgery [12-14]. The decision to await symptom presentation to inform intervention is not trivial as cases have been reported featuring a delayed onset of symptom presentation up to seven years post-procedurally [15-17]. In these cases, surgery was required to remove the emboli after symptomatic presentation.

An individualized approach is warranted for each asymptomatic patient based on a multidisciplinary, diligent risk-benefit analysis considering the location of cement, shape of cement, and comorbidities of the patient. The decision against embolectomy in the patient was determined by the lack of symptoms and a risk-benefit analysis opposing retrieval. Despite the pertinent history of PVC, the patient remains both asymptomatic and unchanged since the procedure. Concerns about the exacerbated risk of thrombosis due to arrhythmia have been previously studied with conflicting evidence that cardiac cement acts as a nidus for thrombogenicity, debating the utility and timing of multimonth anticoagulant usage after the discovery of cardiac cement [16,18]. Anticoagulant therapy is often recommended for a three- to six-month period to reduce this risk during the initial stages of cement endothelialization; however, the individual must be considered as a whole. This patient was very frail and suffered frequent falls with significant injuries. Thus, the risk-benefit of anticoagulation was felt to be adverse, particularly as any resultant thrombi would be right-sided. Given her history of ectopy and recurrent syncope, the rare potential for induction of malignant arrhythmias was assessed by an implantable rhythm monitor to detect the syncope/rhythm correlation and to determine the burden of ectopy. Premature atrial beats and frequent PVC were specifically observed, which were not changed or worsened following the cement embolus. To reduce the risk of potential morbidity, the patient has been recommended for continued surveillance with regular imaging. Given the lack of change of baseline cardiac disease and the patient's reluctance for surgery, the patient has been recommended yearly follow-up with cardiology.

Leakage can be avoided using higher viscosity cement and implanting cement at the latest point in its curing timeframe [1]. In this patient, there was previous extravasation during the prior placement of spinal fusion hardware. Extra care should be taken in cases like this as there is potentially a proclivity for repeated venous uptake of cement. Additionally, the cement used in the upper thoracic spine poses an added risk due to its closer proximity to cardiac return circulation and smaller vertebral body volume. Leakage was found to be three times more likely at these levels [19]. Specific fracture anatomy, particularly endplate cortical dysfunction, has been associated with an increased occurrence of cement leakage, and patients treated for multiple levels in one encounter have been associated with an increased occurrence of cardiac cement embolization [3,20].

## Conclusions

This case presents a kyphoplasty with asymptomatic cardiac and pulmonary artery cement extravasation. All types of cement augmentation can result in leakage with or without symptoms, which has led to varied outcomes in the literature including asymptomatic presentation, conservative medical management, surgical cement retrieval, and sudden death. Thorough imaging, interdisciplinary consultation, and risk-benefit analysis should be conducted to select the best course of action for each patient.
